# Detection of Progression of Glaucomatous Visual Field Damage Using the Point-Wise Method with the Binomial Test

**DOI:** 10.1371/journal.pone.0078630

**Published:** 2013-10-25

**Authors:** Ayako Karakawa, Hiroshi Murata, Hiroyo Hirasawa, Chihiro Mayama, Ryo Asaoka

**Affiliations:** Department of Ophthalmology, University of Tokyo Graduate School of Medicine, Tokyo, Japan; Harvard Medical School, United States of America

## Abstract

**Purpose:**

To compare the performance of newly proposed point-wise linear regression (PLR) with the binomial test (binomial PLR) against mean deviation (MD) trend analysis and permutation analyses of PLR (PoPLR), in detecting global visual field (VF) progression in glaucoma.

**Methods:**

15 VFs (Humphrey Field Analyzer, SITA standard, 24-2) were collected from 96 eyes of 59 open angle glaucoma patients (6.0 ± 1.5 [mean ± standard deviation] years). Using the total deviation of each point on the 2^nd^ to 16^th^ VFs (VF2-16), linear regression analysis was carried out. The numbers of VF test points with a significant trend at various probability levels (p<0.025, 0.05, 0.075 and 0.1) were investigated with the binomial test (one-side). A VF series was defined as “significant” if the median p-value from the binomial test was <0.025. Similarly, the progression analysis was carried out using only second to sixth VFs (VF2-6). The performance of each method was evaluated using the ‘consistency measures’; proportion both significant (PBS): both VF series (VF2-6 and VF2-16) were “significant”, proportion both were not significant (PBNS): both were “not significant”, proportion inconsistently significant (PIS): VF2-16 was “not significant” but VF2-6 was “significant”. A similar analysis was carried out using VF2-7 and VF2-15 series, and the performance was compared with MD trend analysis and PoPLR.

**Results:**

The PBS of the binomial PLR method (0.14 to 0.86) was significantly higher than MD trend analysis (0.04 to 0.89) and PoPLR (0.09 to 0.93). The PIS of the proposed method (0.0 to 0.17) was significantly lower than the MD approach (0.0 to 0.67) and PoPLR (0.07 to 0.33). The PBNS of the three approaches were not significantly different.

**Conclusions:**

The binomial BLR method gives more consistent results than MD trend analysis and PoPLR, hence it will be helpful as a tool to ‘flag’ possible VF deterioration.

## Introduction

Glaucoma is the second most common cause of blindness worldwide[1]. As glaucomatous visual field (VF) damage is irreversible, early detection of impairment is essential to attempt to reduce or halt VF progression by controlling intraocular pressure. One of the most frequently used methods to discriminate progressing from stable VF series is linear regression of the mean deviation (MD) value; this trend analysis is already equipped in the Humphrey Field Analyzer (HFA, Carl Zeiss Meditec, Dublin, CA). However, since the MD value merely averages damage across the entire VF, this approach cannot be sensitive to focal VF progression. Indeed, the clinical usefulness of summary measures, such as MD, has been described as ‘poor’[2,3], due to the fact that summary measures largely ignore the detailed spatial information in VFs and can be insensitive to early localized change[4].

Event analysis is an alternative method to detect VF progression that may be able to detect VF progression earlier than a global trend analysis[5,6]; however, a fundamental caveat of event analysis is that it is not possible to grasp the rate of progression in the VF. In addition, several scoring systems have been proposed to identify VF progression for the purposes of research or clinical trials[7-9], but these are far from being in widespread use in general clinics.

The VF progression analysis software, PROGRESSOR®[10], performs linear regression analysis, in ‘a point by point’ manner. Previous reports have demonstrated the usefulness of this approach for the early detection of VF progression[11-15]. On the other hand, there is a fundamental problem with pointwise linear regression (PLR) , which is its inability to summarize the rate of progression in the whole VF. 

Recently, O’Leary et al have applied a novel approach to summarize the results of PLR; they used permutation analyses of PLR (PoPLR) in an attempt to sum the statistical significance for VF deterioration, according to the individual patient’s data (and its variability)[16]. In this study, we propose a new approach which applies the binomial test to the results of point-wise linear regression (binomial PLR) in order to compare the consistency of this approach with conventional MD trend analysis and PoPLR.

## Subjects and Methods

 The study was approved by the Research Ethics Committee of the Graduate School of Medicine and Faculty of Medicine at the University of Tokyo. Written consent was given by the patients for their information to be stored in the hospital database and used for research. This study was performed according to the tenets of the Declaration of Helsinki.

This retrospective study included 96 eyes from 59 patients (mean ± standard deviation (sd) age: 50.7 ± 12.7 years) with a definitive diagnosis of primary open-angle glaucoma or normal tension glaucoma from the general glaucoma clinic at the University of Tokyo Hospital since 2002. All patients had a minimum of 16 VF tests. Patients were selected from the general glaucoma clinic and initial 16 VFs were analyzed, if a patient had more than 16 VF test results.

Criteria for inclusion were visual acuity better than 6/12, refraction less than 5 dioptre ametropia, no previous ocular surgery (except for cataract extraction and intraocular lens implantation), open anterior chamber angle, and no other posterior segment eye disease. All VFs were recorded using the 24-2 or 30-2 test pattern and the SITA standard strategy with a Goldmann size III target. When the VF was measured using the 30-2 test pattern, only the 52 test points overlapping with the 24-2 test pattern were used for the analysis. Reliability criteria applied were fixation losses less than 25 % and false-positive responses less than 15 %, false-negative rate was not used[17]. Patients who experienced intraocular surgical treatments during the observed period were excluded from the analysis.

Fifteen VFs, after excluding the first VF for learning effects, were used in the analysis. Linear regression was carried out on the total deviation (TD) value of each point on the 2nd to 16th VFs (VF2-16), based on the assumption that progression is well captured after 15 tests. The number of VF test points with a significant slope in the regression analysis was counted for various significance levels: p<0.025, 0.05, 0.075 and 0.1. These were then investigated using the one-sided binomial test, and the median p-value, across all significance levels, was calculated. A VF series was defined as “significant” if the median binomial test p-value was <0.025 (binomial PLR method). Similarly, the progression analysis was carried out using only the 2nd to 6th VFs (VF2-6). In the point-wise method, it is assumed VF damage occurs linearly over time, which is a standard assumption for progression of point-wise VF sensitivity and global VF indices[18,19], [20]. The null hypothesis is that the slope of visual field change is equal to 0. Under the null hypothesis, slope coefficient p-values from linear regression are uniformly distributed from 0 to 1. Consequently, the numbers of test points with p-values less than 0.025, 0.05, 0.075 and 0.1 follow the binomial distribution. If we are to accept the null hypothesis, we would expect the numbers of test points to follow a binomial distribution. However, if the observed numbers deviate significantly from a binomial distribution, the null hypothesis is rejected, and consequently a slope coefficient of zero is considered unlikely to be the result of random chance. The median of these four p-values was taken to determine progression since it is inappropriate to apply Fisher's method [21] to merge p-values when these are correlated; instead, taking the median value is recommended because it can appropriately control for type 1 errors[22]. We defined that a series of VF tests is "significant” if the p-value calculated by binomial PLR is less than 0.025, otherwise, it is “not significant”. The R code for carrying out the binomial PLR is given in the Material S1. The performance of the new method was evaluated using the following ‘consistency measures’: the proportion where both VF series (VF2-6 and VF2-16) were defined as “significant” (‘proportion both significant’; PBS); the proportion where both series were “not significant” (‘proportion both not significant’; PBNS); the proportion where VF2-16 was “not significant” but VF2-6 was “significant” (‘proportion inconsistently significant’; PIS). Thus, in our analysis, the longer VF series represents a surrogate for the ground truth. Consequently, PIS (short series significant and long series significant) is a surrogate measurement for the false positive rate, PBNS (short series not significant and long series not significant) is a surrogate measurement for true negatives and PBS (short series significant and long series significant) is a surrogate measurement for true positives. Then, a conventional trend analysis of MD was carried out, and the consistency measures defined above were evaluated. In addition, the recently-developed PoPLR method was carried out to estimate its performance using the same approach. The analyses were also repeated using different series intervals: VF2-7 and VF2-15.The number of eyes defined as ‘significant’ in at least 3 consecutive VFs that then returned to a “not significant” classification, was compared between the approaches.

The novel binomial PLR approach was compared with the standard trend analysis of MD using a statistical test to perform a pairwise comparison between pairs of proportions, with correction for multiple testing (“pairwise.prop.test” function on the R statistical software). PoPLR was carried out using the R package ‘visualFields’. All statistical analyses were carried out using the statistical programming language R[23] and Medcalc version 11.4.2.0; MedCalc statistical software, Mariakerke, Belgium.

## Results

Subject characteristics are given in Table 1. The average baseline MD was -7.5 (standard deviation (sd): ± 5.3) dB. The observation period (VF2-16) spanned a mean (± sd) of 6.0 (± 1.5) years, and the mean (± sd) rate of progression was -0.37 ± 0.48 dB/year. The VF data used are given in Material S2.

**Table 1:** Subject demographics.

Age, y, mean ± SD	50.7 ± 12.7
Gender (Male : Female)	50 : 46
MD of initial VF, dB, mean ± SD	-7.5 ± 5.3
Type of glaucoma (POAG, NTG, SOAG)	27, 58, 11

**Figure 1:**
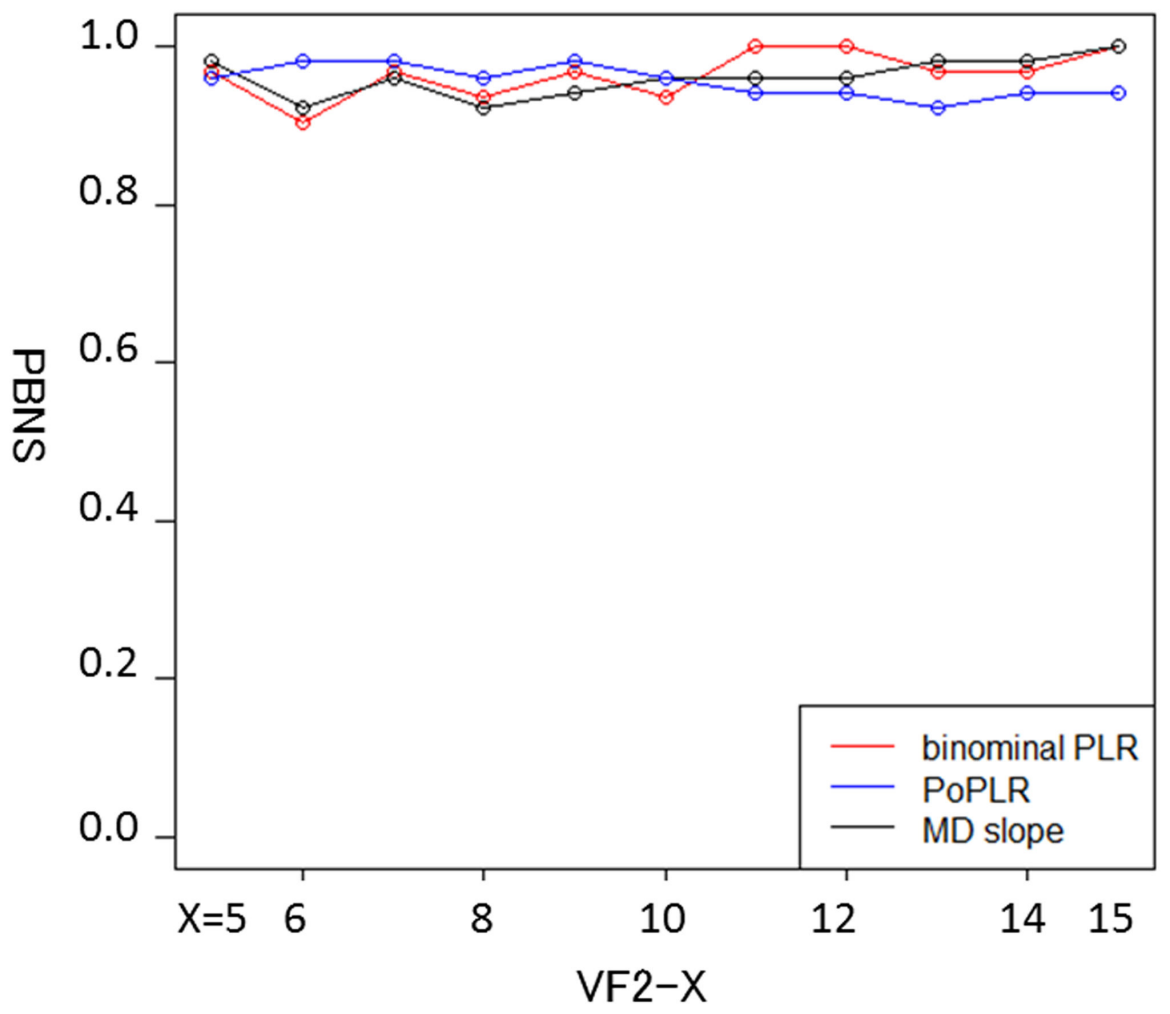
PBNS of detecting progression by MD slope method and point-wise method with the binomial test. PBNS: proportion of VF series where both were not significant, MD: mean deviation.

Age and Mean Deviation are described as mean ± standard deviation. POAG: primary open angle glaucoma, NTG: normal tension glaucoma, SOAG: secondary open angle glaucoma.Figure 1 shows the PBNS for the binomial PLR method, PoPLR method and standard MD trend analysis. The PBNS of the binomial PLR method varied between 0.90 and 1.0 (median: 0.97) depending on the number of VFs in the series, whilst the PBNS of PoPLR and MD trend analysis varied between 0.92 and 0.98 (median: 0.96), and 0.92 and 1.0 (median: 0.96), respectively. There was not a significant difference between the PBNS of the three methods (p > 0.05, Friedman test).

**Figure 2:**
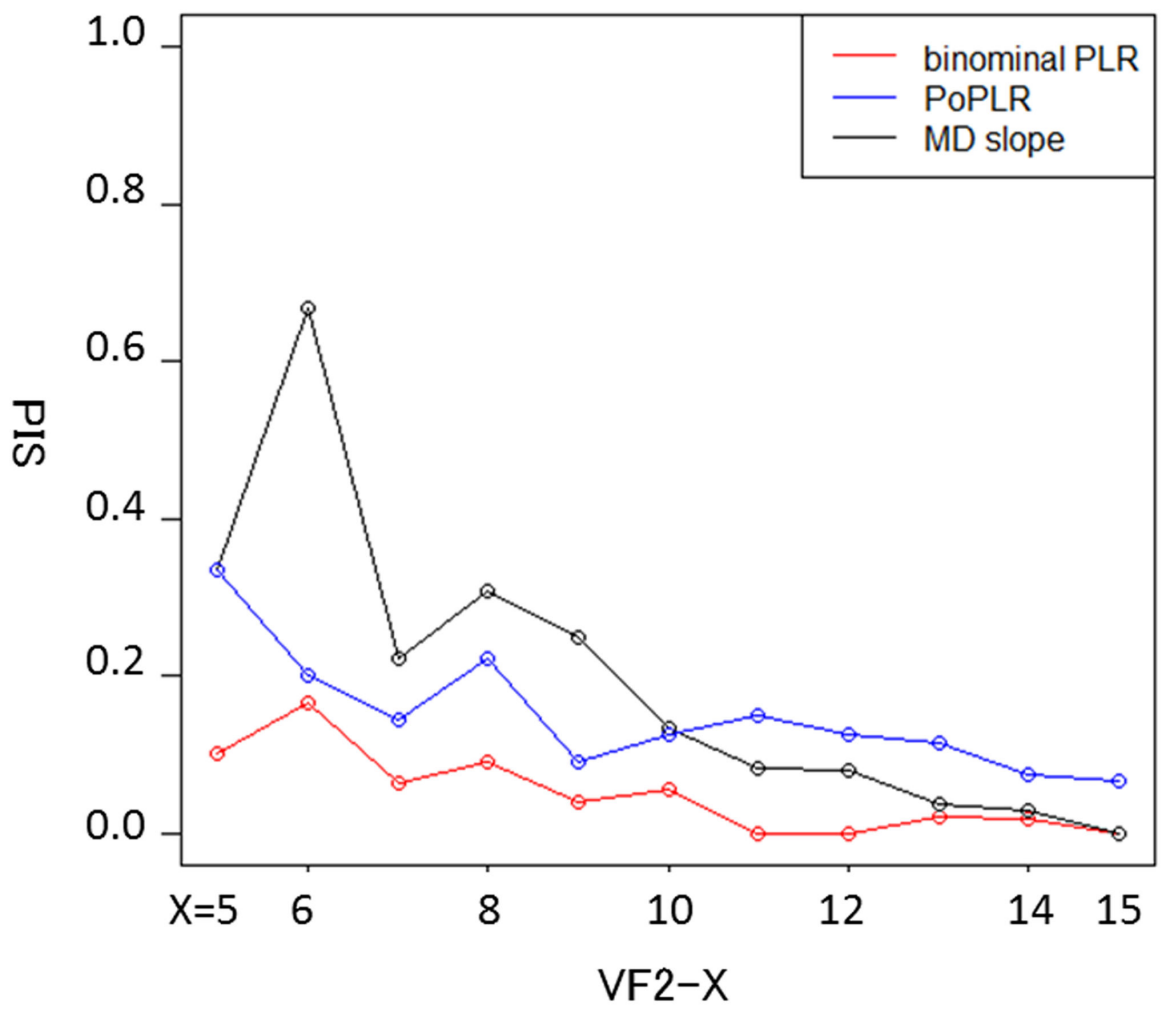
PIS estimate of detecting progression by MD slope method and point-wise method with the binomial test. PIS: proportion of VF series with inconsistent significance, MD: mean deviation.

Figure 2 shows the PIS of the three methods; the rate of the binomial PLR method varied between 0.0 and 0.17 (median: 0.04) depending on the series length, whilst the PIS of the PoPLR and MD trend analysis methods varied between 0.07 and 0.33 (median: 0.13), and 0.0 and 0.67 (median: 0.13), respectively. There was a significant difference between the PIS of binomial PLR and the other two methods (p < 0.05, Friedman test).

**Figure 3:**
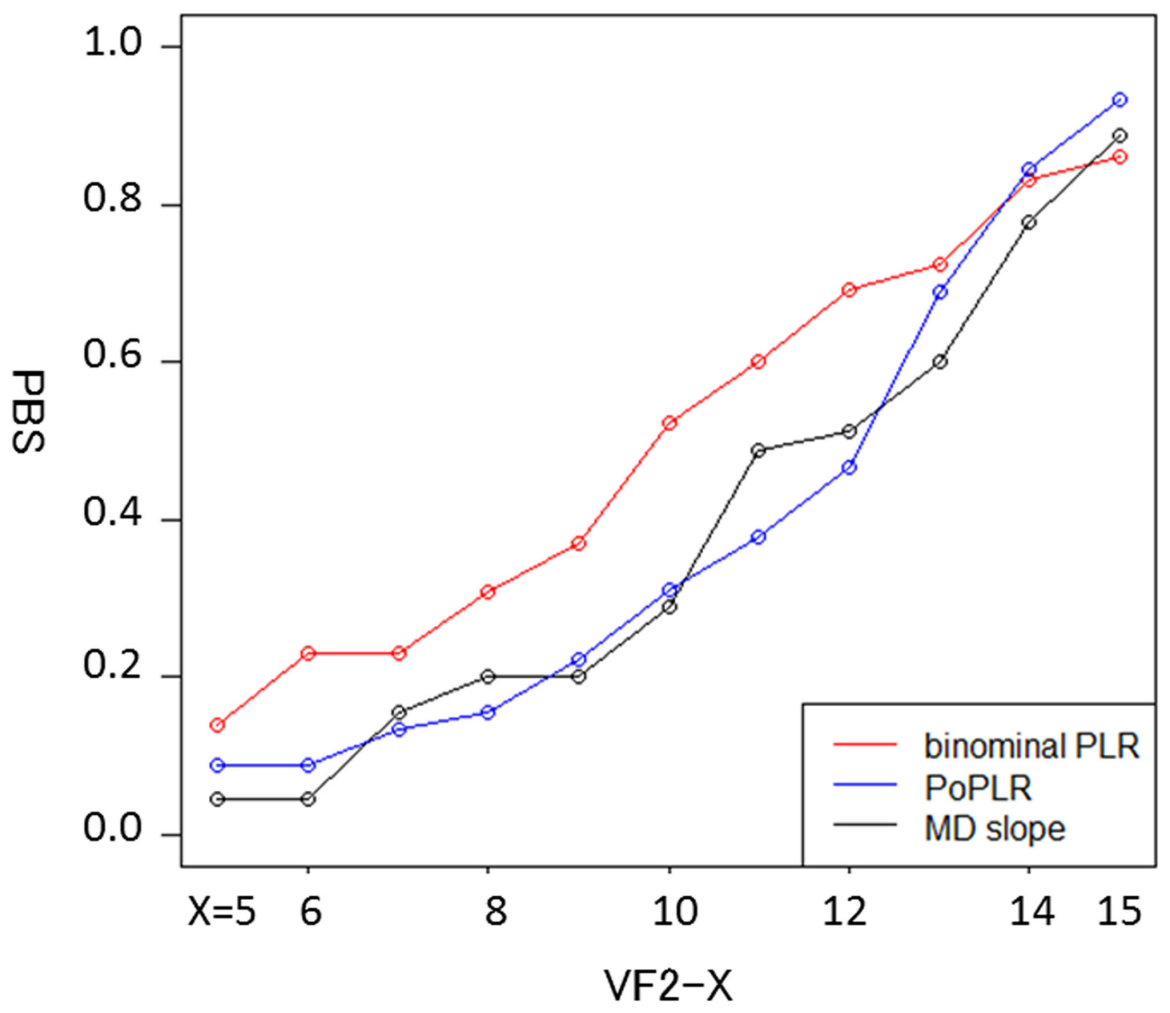
PBS estimate of detecting progression by MD slope method and point-wise method with binomial test. PBS: proportion of VF series where both were significant, MD: mean deviation.

Figure 3 shows the PBS of the proposed binomial PLR method, PoPLR method and standard MD trend analysis. The PBS of the novel binomial PLR method varied between 0.14 and 0.86 (median: 0.52) depending on the series length, whilst the PBS of PoPLR and the conventional MD method varied between 0.09 and 0.93 (median: 0.31), and 0.04 and 0.89 (median 0.29), respectively. There was a significant difference between the PBS values of the binomial PLR method and the other two methods (p < 0.05, Friedman test). As shown in Figures 1 - 3, the three methods tend to converge at the 15th visit, because PBS, PBNS and PIS were calculated against the 16th visit.

 Over the entire series (VF2-16), 49 of the total 96 eyes (51.0%) and 51 eyes of the total 96 eyes (53.1%) were diagnosed as “significant” using the MD trend analysis method and PoPLR respectively. On the other hand, 65 eyes of the total 96 eyes (67.7%) were classified as “significant” using the novel binomial PLR method. There was a significant difference between the MD trend analysis (p = 0.027, pairwise comparison for proportions) and binomial PLR, though no significant difference were observed between PoPLR and binomial PLR (p= 0.055 respectively, pairwise comparison for proportions). Furthermore, all eyes diagnosed as “significant” using the MD slope method and PoPLR were also diagnosed as “significant” with the new binomial PLR approach.

 With the MD trend analysis method, nine out of the 46 eyes classified as “significant” in at least three consecutive VF series returned to a “not significant” classification subsequently; for the proposed binomial PLR approach, the proportion was only eight in 57 eyes. This difference was not significant (p = 0.63, pairwise comparison for proportions).

## Discussion

 In this study, we carried out linear regression for each point in the VF, and applied the binomial test to establish if there was significant progression. This approach was contrasted with the standard method of analyzing linear change in MD and the novel PoPLR method. The PBNS of all methods were high, but the PIS was significantly lower with the new binomial PLR approach, and furthermore, the PBS was significantly higher for the proposed method. It is important to note that the *consistency measures* outlined here are not indicative of the *diagnostic* performance of each method, since we have no ground-truth comparator. However, the binomial PLR method outlined provides a method to flag possible progression with respect to an assumed distribution of stable VF series and therefore may help clinicians provide an accurate diagnosis. 

A limitation of the study is the absence of a gold-standard test for glaucoma progression hence it was necessary to use consistency measures to compare methods. We used the complete series of VFs (VF2–16), taken over roughly six years, to denote ‘true’ progression or not. However, it remains controversial how many VFs are needed to acquire accurate results from point-wise linear regression, although studies suggest the minimum number ranges from five[24] to eight, but sometimes higher[25,26]. These findings suggest that the length of VF series used here is adequate.

 The difference in PIS between the MD approach, PoPLR and the proposed approach is perhaps the most convincing result presented to support the utility of the novel method, since it uses each subject's VF series as a reference. Nevertheless, this highlights a further caveat: the rate of change in VF sensitivity is rarely constant over many years. Thus, it is plausible that a VF could progress rapidly for the first few years, and then much slower after that, especially because clinicians identifying rapid progression would treat these patients more aggressively. Nonetheless, this possibility would affect all methods, and hence the surrogate to evaluate the PIS.

Trend analysis with a global index has been a standard practice since the early days of standard automated perimetry to detect VF change over time[2]. The Glaucoma Progression Analysis (GPA) software in the HFA gives an alert of ‘MD SLOPE SIGNIFICANT’ when the p-value of the MD slope is less than 0.05, hence we used this method as a reference standard in the current study. Previous research has revealed that trend analyses of a global index are conservative and have relatively low sensitivity[2,27,28]. The advantage of the point-wise trend procedure is that spatial information, characterizing focal glaucomatous damage, is preserved. In the current study, we have proposed a novel method to determine global VF progression, which aggregates the results of the point-wise linear regression analysis using the binomial test.

 Recently, O’Leary et al. proposed a novel technique of applying permutation testing to point-wise trend analysis[16]. The current study has commonality in that the results of point-wise linear regression are summarized as an average value. One of the advantages of the current approach is that the binomial test is computationally easy to apply, and is available in all standard statistical software. Furthermore, it has been shown that the binomial PLR approach has a higher PBS value with a lower PIS measurement and without reducing the PBNS measure when compared with PoPLR. Nonetheless, further investigations should be carried out in independent data.

A possible disadvantage of PLR is that specificity may be lost at the expense of increasing sensitivity[28], whereas trend analyses of global indices, such as MD, may have greater specificity but lower sensitivity[29,30]. In the current study, PBNS of the new point-wise method was approximately the same as a trend analysis of MD. Furthermore, the PIS with the proposed approach was significantly lower compared with the standard reference analysis of MD trend analysis. In addition, the proposed method showed an equivalent rate of eyes that returned to a “not significant” trend following at least three consecutive classifications of “significant”. In short, the new binomial PLR method has the merits of both a point-wise linear regression analysis and a trend analysis of a VF summary measure, and displays, high consistency. 

 Nouri-Mahdavi et al. investigated the ‘sensitivity’ of point-wise linear regression against an event analysis method. For the latter approach, the VF was judged as progressing when 3 or more VF points, not necessarily contiguous, demonstrated worsening on at least 3 consecutive visual fields[31]. In the study, the outcome after an 8-year follow-up was considered to be the reference, similarly to the approach in the current study. As a result, the cumulative proportion of progressing eyes was merely 35%, despite the long observation period. The binomial PLR method proposed in the current study obtained a much higher PBS than the aforementioned event analysis in the previous publication, although the difference may be attributed to its stringent definition of progression – worsening needed to be confirmed on at least 3 consecutive VFs, which would take between one to one and a half years (considering the frequency of VF monitoring: 18 VFs were measured over eight years). In the clinical setting, decisions should be made as early as possible to maintain a patient’s visual function and quality of life, since glaucomatous VF damage is irreversible. The proposed binomial PLR approach may help clinicians to discriminate between stable and progressing VFs, because of its high consistency. 

One of the possible caveats with the suggested method concerns the mode of progression. In a previous study, Caprioli et al. suggested that point-wise damage may occur in an exponential fashion[32]. Also, Mikelberg et al. have suggested there episodic progression sometimes occurs (estimated as 7% in their study population) [33]. Nevertheless, other studies have demonstrated that a linear trend analysis can accurately describe VF progression, and predict future damage in the VF[11,34,35]. On the other hand, Rao and colleagues have suggested that the speed of VF progression varies according to the stage of glaucoma[36], so future research would be worthwhile to investigate whether the method proposed in this study is influenced by stage of glaucoma. However, consistency was assessed in the same way for all methods analyzed here, so results are comparable. Furthermore, the binominal test can be applied to summarize the results of any point-wise analysis, as long as a p-value is obtained at each location. 

 Recently, there has been a renewed interest in summary measures of the VF, with the development of the Visual Field Index (VFI)[34]. The VFI is calculated from the pattern deviation probability plot in eyes with a mean deviation (MD) of better than -20 decibels (dB) to reduce the potentially confounding effects of cataract, and many recent studies have used VFI instead of MD to investigate progression[5,37-41]. Thus, it would be interesting to compare the current approach with a VFI trend analysis; however, we do not expect a notable difference since Artes et al. have suggested that a similar percentage of eyes are classified as progressing using the two global indices, and moreover, agreement of a progression classification was good between the two global indices[42]. Furthermore, the VFI calculation uses pattern deviation probability plot values when MD is greater than -20 dB, and total deviation probability plot values when MD is worse than -20 dB, hence it is questionable whether it is appropriate to carry out linear regression if damage spans this threshold[43]. Thus it would be necessary to carry out a future study to compare the VFI trend analysis and binomial PLR approach using the pattern deviation indices.

 Another possible limitation of the current study is the mixture of 30-2 VF and 24-2 VF. This is because fatigue effects are potentially different since more points are tested in the 30-2 VF compared with the 24-2 VF. Nonetheless, the main results should be largely unaffected, because all methods compared share this in common. In addition, to investigate the possible biases of using different number of eyes per patients, similar analyses were carried out using only one eye per patient. As a result, no remarkable differences in the results were observed (data not shown). 

 In conclusion, we have applied the binomial test on the results of point-wise linear regression. The PBS consistency measure of this approach is significantly higher than a conventional trend analysis with MD. Furthermore, the new approach demonstrates a significantly lower PIS without losing consistency in the PBNS measurement.

## Supporting Information

Supplemental Material S1 **R code to perform the binomial test on pointwise linear regression.**
(DOCX)

Supplemental Material S2 **Visual field data analyzed.**
(CSV)
