# Supplementary material for: Detection of Progression of Glaucomatous Visual Field Damage Using the Point-Wise Method with the Binomial Test
Source: PLoS One. 2013 Oct 25;8(10):e78630. doi: 10.1371/journal.pone.0078630 (PMC3808340; doi:10.1371/journal.pone.0078630)
Supplement: Supplemental Material S1 — R code to perform the binomial test on pointwise linear regression. (DOCX) [file pone.0078630.s001.docx]

**#R program to perform binomial PLR analysis.**

#Please note that comments/instructions on the code are preceded by a ‘#’ symbol.

# Specify the directory of visual field data if you need, and the working directory must contain the “data.csv”file.

# Read in data in required format; ‘data.csv’is a CSV file containing IDs, years form the first VF test, and the TD values

# Column headers

#id1: ID for patients

#id2: ID for eyes

#time: years from the first time VF test

#TD_6, TD_7, TD_8, TD_9, TD_12, TD_13, TD_14, TD_15, TD_16, TD_17, TD_20, TD_21, TD_22, TD_23, TD_24, TD_25, TD_26, TD_27, TD_30, TD_32, TD_33, TD_34, TD_35, TD_36, TD_37, TD_38, TD_40, TD_42, TD_43, TD_44, TD_45, TD_46, TD_47, TD_48, TD_50, TD_51, TD_52, TD_53, TD_54, TD_55, TD_56, TD_57, TD_60, TD_61, TD_62, TD_63, TD_64, TD_65, TD_68, TD_69, TD_70, TD_71: total deviation of the 52 VF test points composing 24-2

library(foreach)

Data.VF <- read.csv("data.csv")

#converting column “id2”from numeric values to factors

Data.VF$id2 <- factor(Data.VF$id2)

#index of TD values of test points that are ovelapping with 30-2 and 24-2

#only the 52 test points that compose 24-2 test points are used

index.td.24 <- (1:76)[-c(1:5, 10:11, 18:19, 28:29, 39, 49, 58:59, 66:67, 72:76, 31, 41)]

###############################################################

########function to calculate combined p-values: in accordance with the following article######

# van de Wiel MA, Berkhof J, van Wieringen WN.

#Testing the prediction error difference between 2 predictors.

#Biostatistics. 2009 Jul;10(3):550-60. doi: 10.1093/biostatistics/kxp011. Epub 2009 #Apr 20.

#x: a numeric vector that are calculated by point-wise linear regression

#x: you should specify either “median”or “qnorm”. The detail is described in the aforementioned paper.

#p: a numeric vector that contain thresholds used in the binomial test. The default value is c(0.025, 0.05, 0.075, 0.10).

###############################################################

calc.p <- function(x, method=c("median", "qnorm"), p=seq(0.025, 0.1, by=0.025)) {

p.value <- numeric(length(p))

for(i in 1:length(p)) {

abnormal.num <- sum(x < p[i])

result.binom <- binom.test(abnormal.num, 52, p=p[i], alternative="greater")

p.value[i] <- result.binom$p.value

}

if(method[1] == "median") return(median(p.value))

return(pnorm(mean(qnorm(p.value))))

}

#######################binomial PLR#################################

######predict VF15 from VF 2-4, 2-5, 2-6, 2-7, 2-8, 2-9, 2-10, 2-11, 2-12, 2-13 and 2-14#######

################################################################

result.pointwise.linear.regression <- foreach(k=4:15, .combine="c") %do% {

result <- NULL

for(i in as.character(unique(Data.VF$id2))) {

#create subset of data

data.subset <- Data.VF[Data.VF$id2 == i, ]

#omit the first VF test

data.subset <- data.subset [2:16, ]

#subset of the first k VF tests

data.subset <- data.subset [1:k, ]

#temporary result

result.temp <- NULL

for(j in paste("TD_", index.td.24, sep="")){

lm.result <- summary(lm(data.subset [, j] ~ data.subset $time))

if(is.nan(lm.result$coefficients[2, 4])){

result.temp <- c(result.temp, 0.5)

next

}

if(lm.result$coefficients[2, 1] >= 0) {

result.temp <- c(result.temp, 1 - lm.result$coefficients[2, 4] * 0.5)

next

}

result.temp <- c(result.temp, lm.result$coefficients[2, 4] * 0.5)

}

result <- rbind(result, result.temp)

}

list(result)

}

p.combine <- NULL

for(i in 1:length(result.pointwise.linear.regression)) {

p.combine <- c(p.combine, list(apply(result.pointwise.linear.regression [[i]], 1, function(x) calc.p(x, method="qnorm"))))

}

#table of specificity, sensitivity, and false positive ratio on VF2-5 to VF2-15

performance.binomialPLR <- matrix(NA, nrow=length(p.combine) - 1, ncol=3)

for(i in 1:(length(result.pointwise.linear.regression) - 1)) {

cross.table <- table(p.combine[[i]] < 0.025, p.combine[[length(result.pointwise.linear.regression)]] < 0.025)

#specificity

performance.binomialPLR[i, 1] <- cross.table [1, 1] / sum(cross.table[, 1])

#sensitivity

performance.binomialPLR[i, 2] <- cross.table [2, 2] / sum(cross.table[, 2])

#false positive ratio

performance.binomialPLR[i, 3] <- cross.table [2, 1] / sum(cross.table[2, ])

}

colnames(performance.binomialPLR) <- c("PBNP", "PBP", "PIP")

rownames(performance.binomialPLR) <- paste("VF 2-", 5:15, sep="")
